# Synthesis of new cyclotriphosphazene derivatives bearing Schiff bases and their thermal and absorbance properties

**DOI:** 10.3906/kim-1905-60

**Published:** 2020-02-11

**Authors:** Semih DOĞAN, Süreyya Oğuz TÜMAY, Ceylan MUTLU BALCI, Serkan YEŞİLOT, Serap BEŞLİ

**Affiliations:** 1 Department of Chemistry, Faculty of Science, Gebze Technical University, Gebze, Kocaeli Turkey

**Keywords:** Cyclotriphosphazene, Schiff base, thermal stability, UV absorption, substituent effect

## Abstract

In this study, a series of cyclotriphosphazene derivatives containing a Schiff base (3a–3d) were synthesized by the reactions of hexachlorocyclotriphosphazene (1) with bis-aryl Schiff bases ( **2a** – **2d** ) having different terminal groups (H, F, Cl, and Br). The products ( **3a** – **3d** ) were characterized by elemental and mass analyses, FT-IR, and ^1^ H, ^13^ C, and ^31^ P NMR spectroscopies. Furthermore, the structure of compound **3a** was also determined by X-ray crystallography. The thermal behaviors and the spectral properties of the new cyclotriphosphazene compounds ( **3a** – **3d** ) were investigated and the results were compared in the series.

## 1. Introduction

Heterocyclic systems containing mainly phosphorus, nitrogen, sulfur, and oxygen atoms compose a large class of inorganic compounds and they have applications in various areas [1–5]. The cyclophosphazenes, (NPCl _2_ ) _3,4_ , are among the most important members of these systems [3–7]. Cyclotriphosphazene and its derivatives have particularly great stability thanks to their six-membered ring structures. In addition, their physical and chemical properties can be adjusted via appropriate groups substituted on the phosphorus atoms [6–15].

Schiff bases (imines) are among the most popular classes of organic compounds due to their excellent kinetic and thermodynamic stabilities and potential photoelectric properties [16–21]. Aryl Schiff bases, which contain a classical π-conjugated system, have potential optoelectronic properties [16,17,22,23]. There are important influences on the molecular properties due to the substituents on the aromatic rings of an aryl Schiff base [16,24,25].

Although some cyclophosphazene derivatives containing Schiff bases were randomly synthesized in recent years [8,26–32], their thermal stabilities and absorbance properties have not been investigated systematically. In this study, it was planned to synthesize a thermally stable new type of heterocyclic structures by combining the thermal properties of hexachlorocyclotriphosphazene and Schiff bases. Schiff bases ( **2a** – **2d** ) including H, F, Cl, and Br atoms at the para position on the terminal phenyl ring were prepared and then cyclotriphosphazene (1) was sequentially reacted with the bases. The thermal stabilities of Schiff base-substituted cyclotriphosphazenes ( **3a** – **3d** ) were determined by differential scanning calorimetry (DSC) and thermogravimetric analysis (TGA) techniques, while their absorbance spectral properties were investigated in different solvent systems by UV-Vis spectrometry. Thermal analysis results demonstrated that the thermal stability of the cyclotriphosphazenes containing Schiff bases was significantly higher than that of corresponding Schiff bases. Their UV absorption spectra, which were measured in different solvent systems, showed that λmax of compounds from **3a** – **3d** shifts to the red region.

## 2. Experimental

### 2.1. Materials and physical measurements

Hexachlorocyclotriphosphazene (Aldrich) was purified by fractional crystallization from n-hexane. Dichloromethane (DCM; Merck), n-hexane (Merck), n-heptane (Merck), absolute ethanol (Merck), 4-aminophenol (Aldrich), benzaldehyde (Aldrich), 4-fluorobenzaldehyde (Aldrich), 4-chlorobenzaldehyde (Aldrich), and 4-bromobenzaldehyde (Aldrich) were used as received. Tetrahydrofuran (THF; Merck) was distilled over a sodium/potassium alloy under an atmosphere of dry argon. For sodium hydride with 60% dispersion in mineral oil (Merck, prior to use the oil was removed by washing with dry n-hexane followed by decantation. All reactions were performed under a dry argon atmosphere. CDCl _3_ and dimethyl sulfoxide (DMSO-d _6_ ) for NMR spectroscopy were obtained from Merck. Analytical thin-layer chromatography (TLC) was performed on Merck silica gel plates (Merck, Kieselgel 60, 0.25 mm thickness) with F254 indicator. Elemental analyses were obtained using an ELEMENTAR Vario MICRO Cube. Mass analyses were recorded on a Bruker MALDI-TOF (matrix-assisted laser desorption/ionization-time-of-flight) spectrometer using 1,8,9-trihydroxyanthracene as a matrix for compounds **3a** – **3d** . Infrared spectra were recorded on a PerkinElmer Spectrum 100 Two spectrometer with ATR in the region of 4000–650 cm ^-1^ . ^1^ H, ^13^ C, and ^31^ P NMR spectra were recorded for all compounds in CDCl _3_ for **3a** and **3b** and DMSO-d _6_ for 3c and **3d** on a Varian INOVA 500 MHz spectrometer using TMS as an internal reference for ^1^ H and ^13^ C and 85% H3 PO4 as an external reference for ^31^ P NMR measurements. Electronic absorption spectra in solution were recorded using a Shimadzu 2101 PC UV spectrophotometer. The thermal analyses were performed on Mettler Toledo TGA/SDTA 851 and Mettler Toledo DSC 822 devices. The TGA were measured between 25◦ C and 700◦ C with 10◦ C/min and under 50 mL/min N2 flow. The DSC data were recorded between room temperature (RT) and 300◦ C. The heating rate was also 10◦ C/min.

### 2.2. X-ray crystallography

Intensity data were recorded on a Bruker APEX II QUAZAR diffractometer using monochromated Mo K _α_ X-radiation (λ = 0.71073 Å). Absorption correction was performed by the multiscan method implemented in SADABS [33] and space groups were determined using XPREP implemented in APEX2 [34]. Structures were determined using the direct methods procedure in SHELXS-97 and refined by full-matrix least squares on F ^2^ using SHELXL-97 [35]. All nonhydrogen atoms were refined with anisotropic displacement factors and C-H hydrogen atoms were placed in calculating positions and allowed to ride on the parent atom. The final geometrical calculations were carried out with the PLATON [36] and MERCURY [37] programs and the molecular drawings were done with the DIAMOND [38] program. Structure determination was deposited with the Cambridge Crystallographic Data Centre with reference CCDC 1865974 for compound **3a** . Although the synthesis of compound **3a** exists in the literature [26], the molecular structure of compound **3a** has been determined by X-ray crystallography for the first time in this study.

### 2.3. Synthesis

#### 2.3.1. Synthesis of Schiff bases ( **2a** – **2d** )

The preparations of Schiff bases ( **2a** – **2d** ) were as reported in the literature [16,39]. Schiff bases were prepared by the condensation of 0.1 mol of the corresponding benzaldehyde and 0.1 mol of 4-aminophenol in ethanol for 3 h under reflux. The products crystallized while cooling and were recrystallized from ethanol several times for purification.

#### 2.3.2. General procedure used for synthesis of compounds **3a** – **3d**

Compound **2a** (2.13 g; 10.8 mmol) was dissolved in 10 mL of dry THF in a 100 mL three-necked round-bottomed flask and NaH (60% oil suspension, 0.45 g; 10.8 mmol) in 5 mL of dry THF was quickly added dropwise to the stirred solution under an argon atmosphere. Then a solution of hexachlorocyclotriphosphazene (1) (0.40 g; 1.2 mmol) in 5 mL of THF was prepared and added by dropping funnel dropwise to the stirred solution of **2a** . The reaction was stirred under reflux for 48 h. The reaction was followed by TLC on silica gel plates using n-hexane-THF (3:2) as the mobile phase. The reaction mixture was filtered to remove the sodium chloride and any other insoluble material. The excess THF was removed under reduced pressure and then the reaction mixture was extracted with DCM/distilled water phase for separation from excess Schiff base. n-Hexane was slowly added to the solution and the solids precipitated. The resulting solids were filtered, washed with n-hexane, and then dried at room temperature. Compound **3a** was crystallized from the n-heptane-THF (2:1). A summary of the preparation of all compounds is given in Table 1.

**Table 1:** Preparation of compounds **3a** – **3d** . ^a,b^

Compound 1		Schiff base			Sodium hydride		Yield %	Mp (◦C)	Isolated Cmp
g	mmol		g	mmol	g	mmol			
0.40	1.20	**2a**	2.13	10.80	0.45	10.80	66	172	**3a**
0.40	1.20	**2b**	2.32	10.80	0.46	10.80	61	211	**3b**
0.40	1.20	**2c**	2.50	10.80	0.47	10.80	65	239	**3c**
0.40	1.20	**2d**	2.98	10.80	0.48	10.80	62	262	**3d**

^a^ All reactions carried out for 48 h under reflux. ^b^ n-Hexane/THF (3:2) solvent system was used as mobile phase for TLC analysis.

Anal. calc. for **3a** ; C _78_ H _60_ N _9_ O _6_ P _3_ : C, 71.39; H, 4.61; N, 9.61%, M, 1312.3. Found: C, 71.59; H, 4.68; N, 9.18%, [M+H] ^+^ , 1313.2. FT-IR (cm ^-1^ ) 3059 (Ar-C-H), 1626 (HC=N), 1497 (C=C), 1168, 1202 (P=N), 951 (P-O-C-aryl). ^1^ H NMR, CDCl _3_ , 298 K, δ (ppm) 8.50 (s, H; HC=N), 7.91 (d, 2H, aryl), 7.49 (t, 3H, aryl), 7.21 (d, 2H, aryl), 6.88 (d, 2H, aryl). ^13^ C NMR, CDCl _3_ , 298 K, δ (ppm) 167.28, 160.20, 148.79, 134.45, 129.00, 128.83, 128.68, 121.93, 121.68. ^31^ P NMR, CDCl _3_ , 298 K, δ (ppm) 9.46.

Anal. calc. for **3b** ; C _78_ H _54_ F _6_ N _9_ O _6_ P _3_ : C, 65.96; H, 3.83; N, 8.88 %, M, 1420.3. Found: C, 66.18; H, 3.91; N, 8.27%, [M+H] ^+^ , 1421.5. FT-IR (cm ^-1^ ) 3023 (Ar-C-H), 1627 (HC=N), 1506 (C=C), 1164, 1174 (P=N), 953 (P-O-C-aryl). ^1^ H NMR, CDCl _3_ , 298 K, δ (ppm) 8.32 (m, H; HC=N), 7.78 (m, 2H, aryl), 7.07–7.03 (m, 4H, aryl), 6.87 (m, 2H, aryl). ^13^ C NMR, CDCl _3_ , 298 K, δ (ppm) 165.57, 163.54, 157.07, 154.37, 144.70, 132.67, 130.58, 122.34, 116.00, 115.94, 155.82. ^31^ P NMR, CDCl _3_ , 298 K, δ (ppm) 9.38.

Anal. calc. for **3c** ; C _78_ H _54_ C _16_ N _9_ O _6_ P _3_ : C, 61.68; H, 3.58; N, 8.30%, M, 1519.0. Found: C, 61.75; H, 3.67; N, 7.87%, [M+H] ^+^ , 1518.6. FT-IR (cm ^-1^ ) 3040 (Ar-C-H), 1625 (HC=N), 1494 (C=C), 1168, 1180 (P=N), 949 (P-O-C-aryl). ^1^ H NMR, DMSO-d _6_ , 298 K, δ (ppm) 8.45 (s, H; HC=N), 7.80 (d, 2H, aryl), 7.42 (d, 2H, aryl), 7.14 (d, 2H, aryl), 7.06 (d, 2H, aryl). ^13^ C NMR, DMSO-d _6_ , 298 K, δ (ppm) 158.53, 154.66, 137.50, 130.90, 129.86, 129.46, 128.99, 121.89, 121.67, 115.66. ^31^ P NMR, DMSO-d _6_ , 298 K, δ (ppm) 9.44.

Anal. calc. for **3d** ; C _78_ H _54_ Br6 N _9_ O _6_ P _3_ : C, 52.46; H, 3.05; N, 7.06%, M, 1785.7. Found: C, 52.87; H, 3.14; N, 6.88%, [M+H] ^+^ , 1785.6. FT-IR (cm ^-1^ ) 3033 (Ar-C-H), 1624 (HC=N), 1496 (C=C), 1165, 1200 (P=N), 951 (P-O-C-aryl). ^1^ H NMR, DMSO-d _6_ , 298 K, δ (ppm) 8.44 (s, H; HC=N), 7.72 (d, 2H, aryl), 7.57 (d, 2H, aryl), 7.14 (d, 2H, aryl), 7.05 (d, 2H, aryl). ^13^ C NMR, DMSO-d _6_ , 298 K, δ (ppm) 158.39, 135.51, 133.92, 131.73, 130.05, 125.36, 121.90, 121.47, 113.39. ^31^ P NMR, DMSO-d _6_ , 298 K, δ (ppm) 9.43.

## 3. Results and discussion

### 3.1. Synthesis and characterization of compounds

**Scheme 1:**
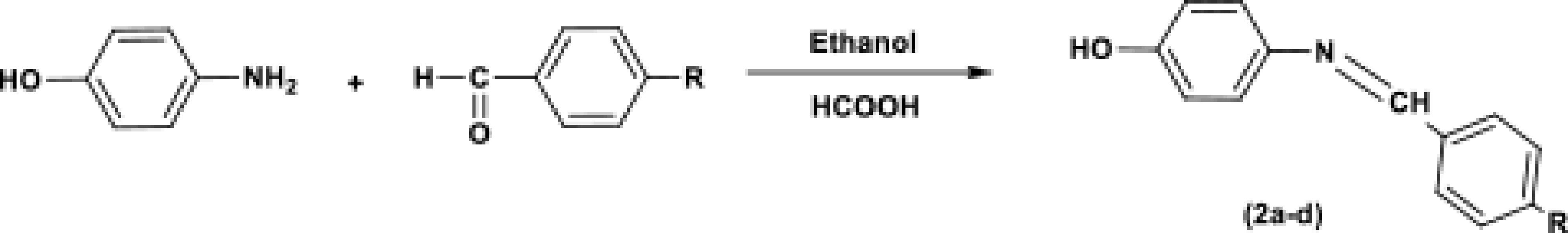
The synthesis pathway of Schiff bases **2a** Ð- **2d** .

**Scheme 2:**
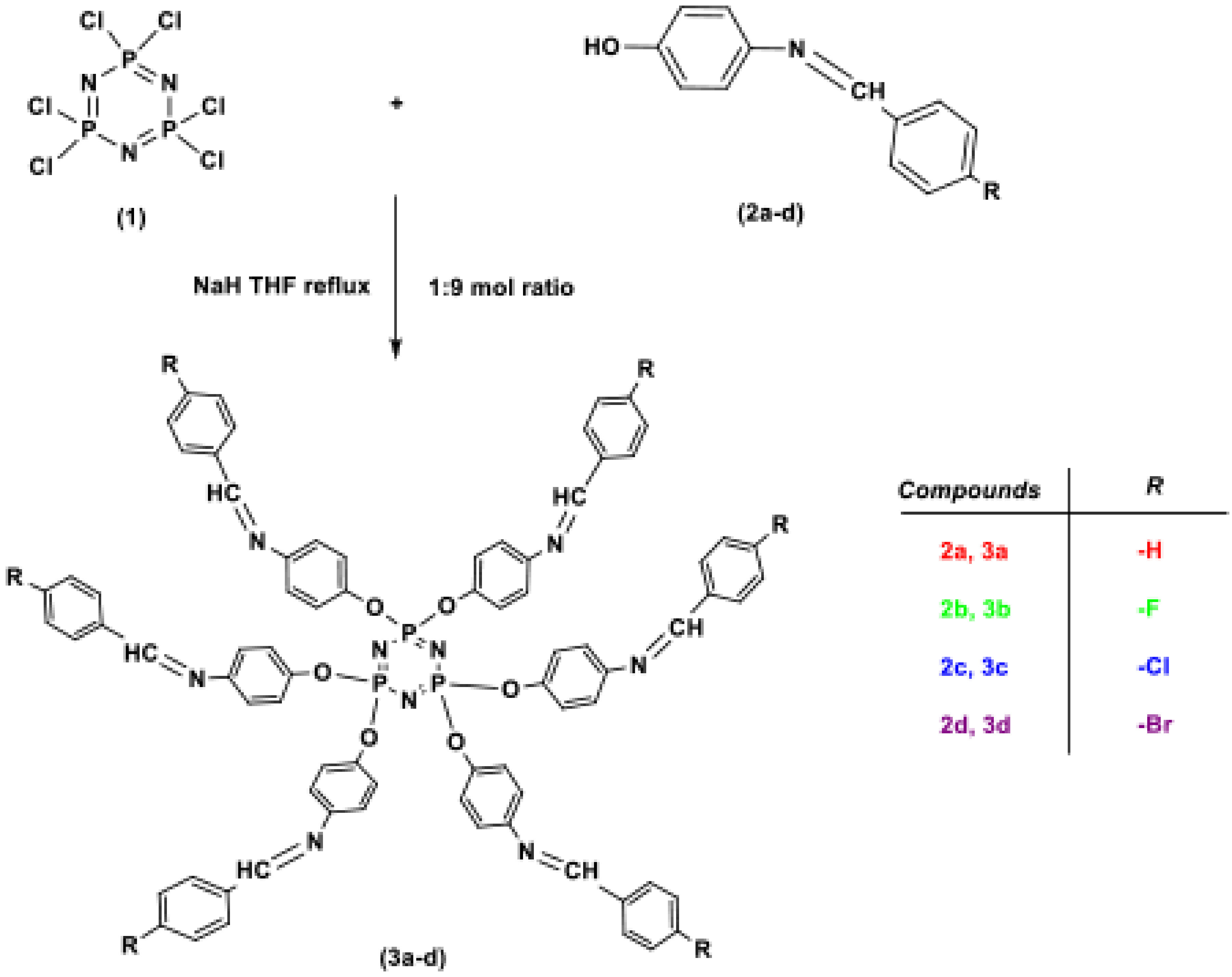
The synthesis pathway of compounds **3a** Ð- **3d** .

Schiff base ligands ( **2a** – **2d** ) bearing four different side groups at the para position on the terminal phenyl ring were prepared from the condensation reaction of 4-aminophenol with benzaldehyde, p-fluoro-, chloro-, and bromobenzaldehyde in ethanol [16,39]. Full substituted cyclotriphosphazene products ( **3a** – **3d** ) were synthesized from the nucleophilic substitution reactions of chlorocyclotriphosphazene (1) with Schiff bases including hydroxyl groups in high yields. The general synthesis route and the structures of compounds **3a** – **3d** are shown in Scheme 1 and Scheme 2. All compounds ( **3a** – **3d** ) were characterized by FT-IR, elemental analysis, MALDITOF mass spectrometry, and ^1^ H, ^13^ C, and ^31^ P NMR spectroscopies. The molecular structure of compound **3a** was also determined by X-ray crystallography. The analysis of the data for each new compound is provided as part of the analytical data in Section 2.3.

**Figure 1:**
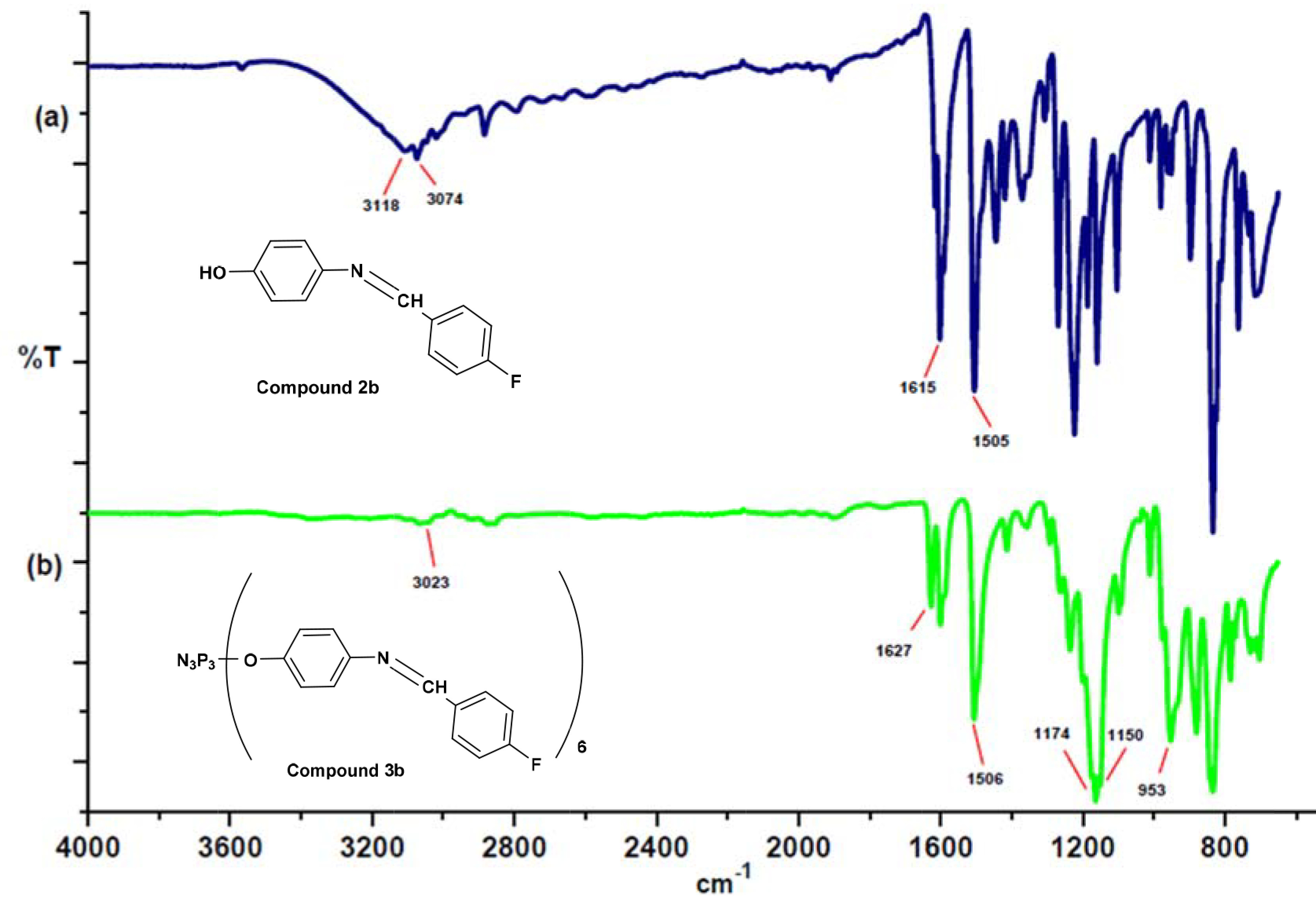
FT-IR spectra of a) compound **2b** and b) compound **3b** .

The FT-IR spectra of compounds **3a** – **3d** showed characteristic stretching bands at 3023–3059 cm ^-1^ (aromatic C–H groups), 1624–1627 cm ^-1^ (C=N stretching vibrations), 1494–1506 cm ^-1^ (alkenyl C=C stretch), 1150–1202 cm ^-1^ (P=N), and 949–953 cm ^-1^ (P–O–C), as expected. The FT-IR spectra of compounds **2b** and **3b** are given as an example in Figure 1. When the IR spectrum of compound **2b** was compared with that of compound **3b** , it was seen that the OH vibration in the 3103–3126 cm ^-1^ region disappeared in the spectrum of compound **3b** and a new band appeared in the 949–953 cm ^-1^ region because of P–OAr absorption. The characteristic P=N stretching vibrations of cyclophosphazenes were observed between 1164 and 1174 cm ^-1^ as sharp bands.

**Figure 2:**
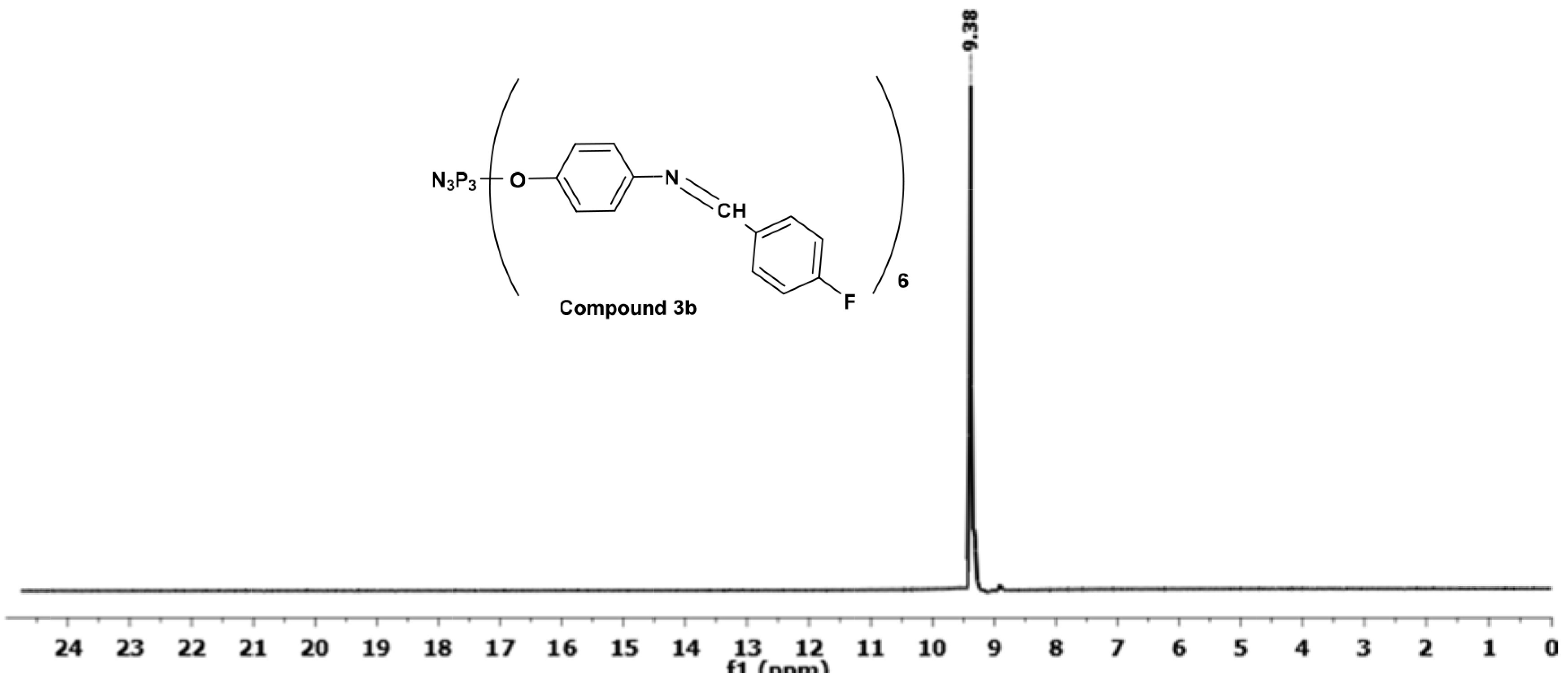
Proton decoupled ^31^ P NMR spectrum of compound **3b** .

The proton decoupled ^31^ P NMR spectra of compounds **3a** – **3d** exhibit an A3 type spin system due to same phosphorus environments within the molecule, as expected, as shown in Supplementary Figures S1–S3. The ^31^ P NMR spectrum of compound **3b** , shown as an example in Figure 2, exhibited a unique sharp singlet at 9.38 ppm.

**Figure 3:**
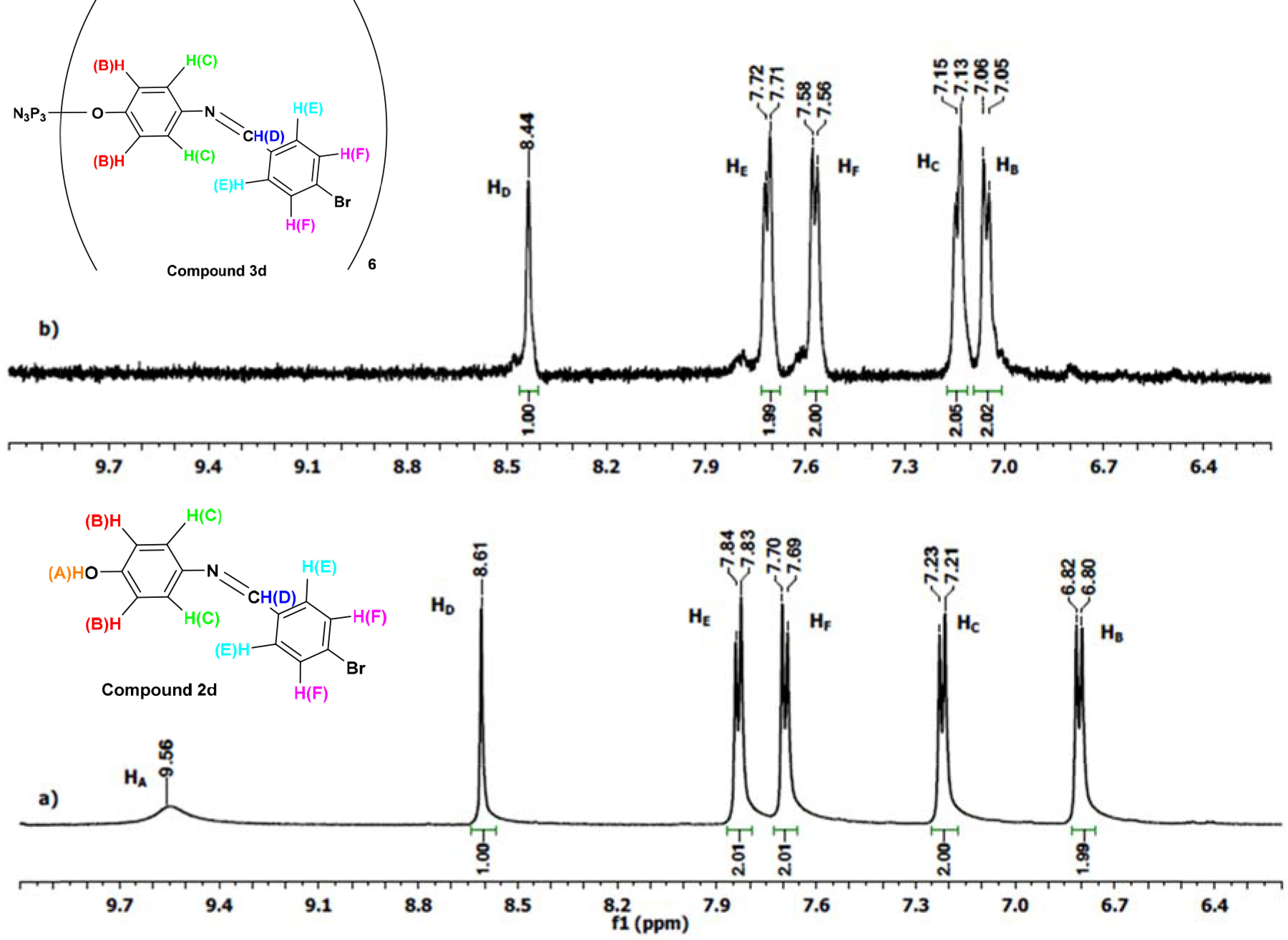
^1^ H NMR spectra of a) compound **2d** and b) compound **3d** in d6 -DMSO.

The ^1^ H NMR spectra of compounds **2a** – **2d** and **3a** – **3d** have similar chemical shifts, except for some obvious differences. For example, while OH protons were observed at 9.53–9.57 ppm for compounds **2a** – **2d** , the signals disappeared in the ^1^ H NMR spectra of compounds **3a** – **3d** . The azomethine protons for compounds **3a** – **3d** were observed between 8.32 and 8.50 ppm as singlets in the spectra. The aromatic protons were observed between 6.87 and 7.91 ppm. The ^1^ H NMR spectra of compounds **2d** and **3d** are given as examples in Figure 3.

**Figure 4:**
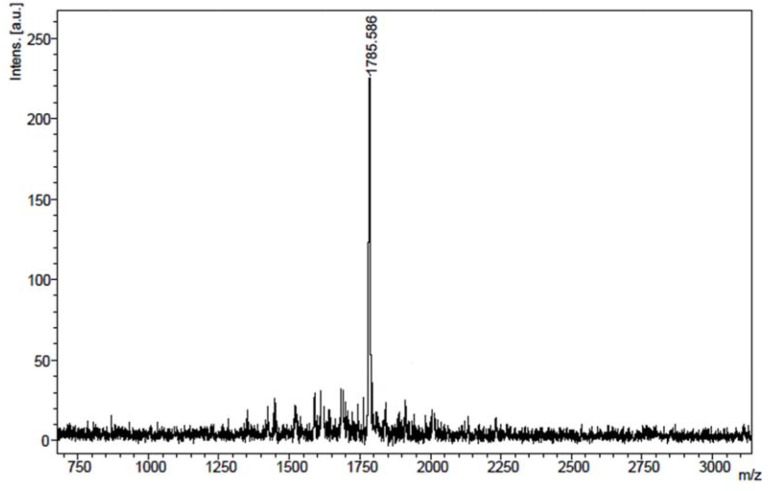
Mass spectrum of compound **3d** .

The MALDI-TOF mass spectra of **3a** – **3d** provided definitive characterization and the results are given in Section 2. The mass spectrum of compound **3d** is given as an example in Figure 4.

### 3.2. Characterization of compound **3a** by X-ray crystallography

**Figure 5:**
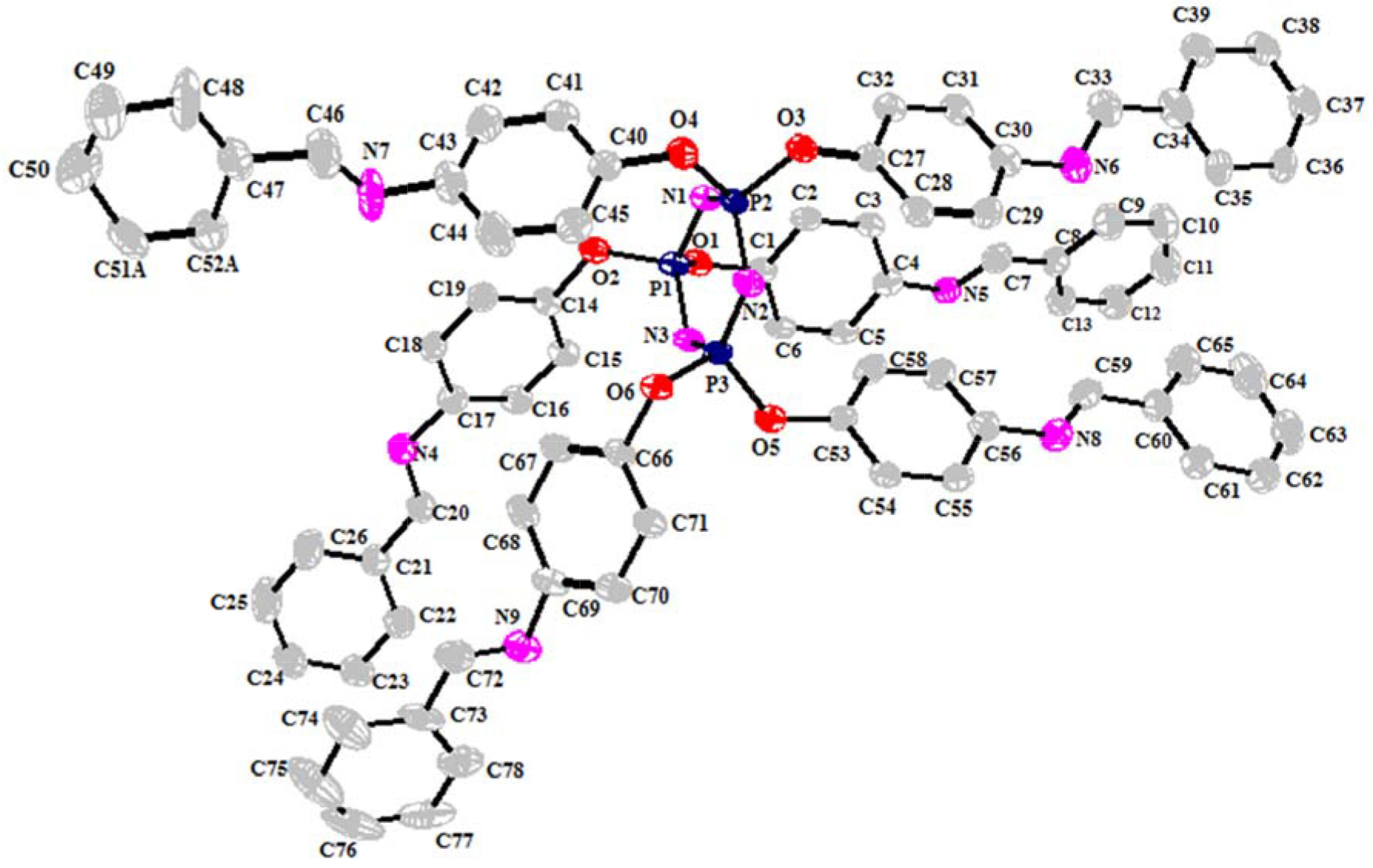
A view of the molecular structure for **3a** with the atom-numbering scheme. Displacement ellipsoids are drawn at the 50% probability level. The hydrogen atoms have been omitted and only one orientation of the disordered C51 and C52 carbon atoms of compound **3a** has been presented for clarity.

The molecular structure of compound **3a** was characterized by X-ray crystallography and its crystal structure is shown in Figure 5. The crystallographic data and refinement parameters are summarized in Table 2. Selected bond lengths and angles are given in Table S1. The molecular structure of compound **3b** (each terminal phenyl ring contains fluorine atoms at the para position) was also confirmed by X-ray analysis, as shown in Figure S4, but the crystal structure could not be fully elucidated due to crystallographic problems. Although different crystallization systems and methods were tried, quality crystals could not be obtained.

**Table 2:** Crystal data and structure refinement details for compound **3a** .

Compound	**3a**
Empirical formula	C _78_ H _60_ N _9_ O _6_ P _3_
Formula weight	1312.26 g/mol
Temperature (K)	120 (2)
Crystal system	Monoclinic
Space group	C 1 2/c 1
a (Å)	46.790 (4)
b (Å)	7.9041 (6)
c (Å)	39.079 (3)
αα◦)	90◦
ββ (◦)	114.969 (4)◦
γγ◦)	90◦
Volume/Å−3	13101.9 (18)
Z	8
Density calc./g cm−3	1.331
μ/mm−1	0.155
F(000)	5472
Crystal size/mm3	0.18 × 0.24 × 0.30
Radiation	Mo K _α_ (λ = 0.71073 Å)
2θ range for data collection/◦	2.94 to 25.00◦
Completeness	0.992
Index ranges	–55 ≤ h ≤ 53, –9 ≤ k ≤ 9, –46 ≤ l ≤ 46
Reflections collected	74825
Independent reflections	11463 [R (int) = 0.0546]
Data/restraints/parameters	11463 / 66 / 872
Goodness-of-fit on F ^2^	1.044
FinalR indexes [I ≥ 2σ(I)]	R1 = 0.0654, wR2 = 0.1560
Final Rindexes [all data]	R1 = 0.0971, wR2 = 0.1690
Largest diff. peak/hole/e Å−3	0.758 and –0.562

Compound **3a** is crystallized in the monoclinic space group C 2/c, with a = 46.790 (4) Å; b = 7.9041 (6) Å; c = 39.079 (3) Å; α, γ = 90◦ ; and β = 114.969 (4)◦ (shown in Table 2). The phosphazene consists of a six-membered ring, (PN) _3_ , which is substituted with six bis-aryl Schiff base groups on the three P atoms. Three of the six arms are located on one side of the ring, while the others are on the other side. The six-membered C _16_ P _3_ ring is a very slightly twisted conformation in the compound with the maximum deviation from the plane of the cyclotriphosphazene ring being only 0.066 Å (on P2 atom) with a total puckering amplitude QT of 0.154 Å, as shown in Figure S5 and Table S1. Normally, P-N bond lengths in the hexachlorocyclotriphosphazene ring are equal to each other and the P-N bond distance is 1.581 Å. N-P-N and P-N-P bond angles are 118.40◦ and 121.40◦ , respectively. In compound **3a** , the average values of endocyclic bond parameters of the phosphazene ring are 1.578 Å for the P-N bond distance;nd 117.19◦ for the N-P-N angle. The P-N-P bond angles are a little larger than the N-P-N bond angles and the average value of P-N-P angles is 121.55◦ (Table S1). Thus, the P-N bond lengths are in the normal ranges and all of these bond parameters were compatible with those reported for fully substituted cyclotriphosphazene derivatives [29,40,41].

The investigation of the crystal structure of compound **3a** shows that there is no classical hydrogen bond but there are many short intermolecular contacts where separation between donor and acceptor atoms is less than 3.5 Å. These contacts are observed predominantly between the hydrogen atoms and heteroatoms (C, N , O) . The orientations of the phenyl rings allow intermolecular π-π interactions, which may be effective in the stabilization of this self-organization. The intramolecular π-stacking interaction is 4.409 Å in the solid state, as shown in Figure S6.

### 3.3. Thermal properties

Cyclotriphosphazene, consisting of alternating phosphorous and nitrogen atoms, exhibits unusual thermal properties such as flame retardancy and self-extinguishing ability [42–44]. Additionally, it can be decorated with thermally and chemical stable groups via nucleophilic substitution reactions in order to further increase its thermal stability. Here, the cyclotriphosphazene ring was appended with Schiff bases, which are also attractive compounds owing to cheapness, ease of synthesis, and their chemical and thermal stability.

**Figure 6:**
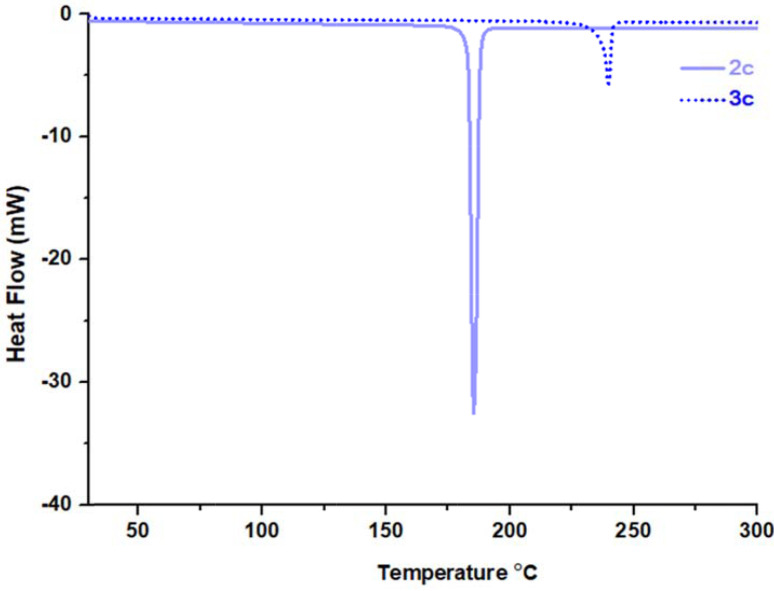
The DSC curves of compounds **2c** and **3c** heated under nitrogen to 300 ◦ C at a heating rate of 10◦ C/min.

**Figure 7:**
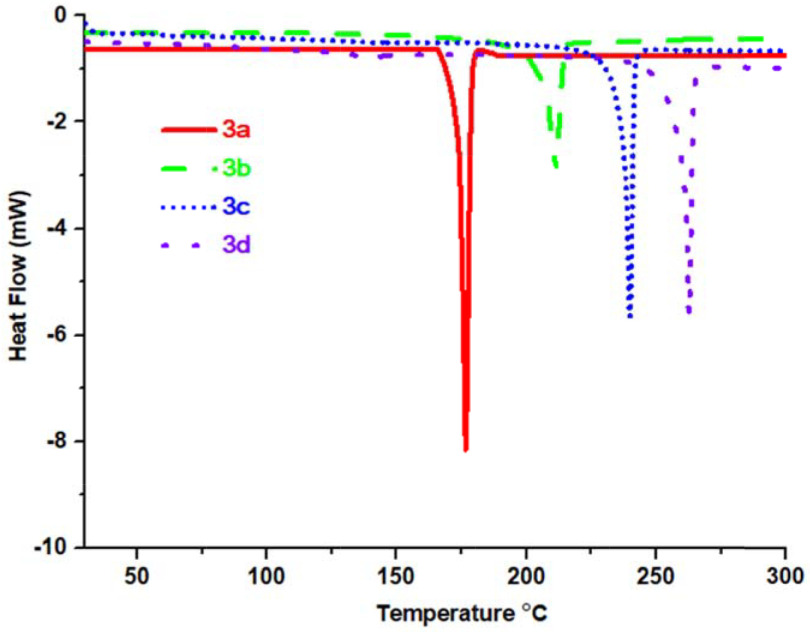
The DSC curves of compounds **3a** – **3d** heated under nitrogen to 300 ◦ C at a heating rate of 10 ◦ C/min.

The melting points (Tm) of all compounds ( **2a** – **2d** and **3a** – **3d** ) were determined by DSC technique and are shown in Figure 6 and Figures S7–S9. The endothermic sharp peak belonging to the melting point in the DSC thermograms of compounds **3a** – **3d** showed that the compounds were obtained in high purity, as shown in Figure 6. DSC analysis results indicated that the melting points of compounds **3b** , **3c** , and **3d** were higher than those of corresponding Schiff base compounds **2b** , **2c** , and **2d** , excluding **3a** , as shown in Table 3. When the DSC curves given in Figure 7 are examined, it can be said that the melting point increased in the order of terminal groups, which is hydrogen, fluorine, chlorine, and bromine.

**Table 3:** Thermal analysis results of compounds 1, **2a** – **2d** , and **3a** – **3d** .

Compounds	DSC (oC) (Tm)	First mass loss in TGA (◦C)		Remaining amount at 700 ◦C (%)
		T _on_	T _dm_	
Cyclotriphosphazene				
1	114	159	167	1.4
Schiff bases				
**2a**	187	206	275	14.5
**2b**	153	198	290	19.6
**2c**	184	234	292	38.5
**2d**	207	234	280	38.6
Cyclotriphosphazene derivatives				
**3a**	175	398	458	62.5
**3b**	211	366	427	56.5
**3c**	239	379	431	56.6
**3d**	262	396	436	62.9

T _m_ : Melting temperature. T _on_ : Onset temperature, starting point of the decomposition processes. T _dm_ : Maximum point of decomposition temperature.

**Figure 8:**
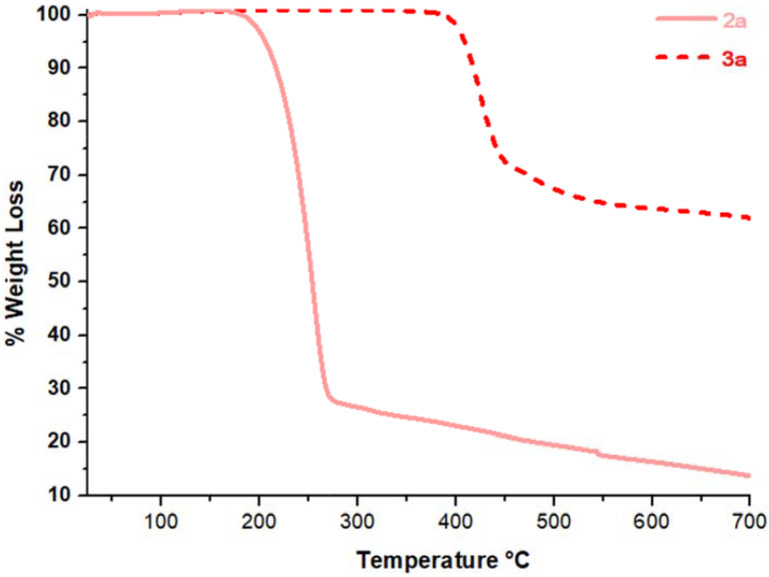
TGA curves of compounds **2a** and **3a** from 25 ◦ C to 700 ◦ C at a heating rate of 10 ◦ C/min under N2 flow of 50 mL/min.

TGA thermograms of the compounds ( **2a** – **2d** and **3a** – **3d** ) were recorded between RT and 700◦ C under 50 mL/min N2 flow. TGA was utilized to evaluate the thermal stability of the Schiff bases ( **2a** – **2d** ) and compounds ( **3a** – **3d** ). The onset decomposition temperatures (T _on_ ) were recorded at a heating rate of 10◦ C/min. The curves of the TGA measurements are given in Figures S10–S12. The onset decomposition temperatures (T _on_ ) , maximum point of decomposition temperatures (T _dm_ ) , and remaining substance amounts at 700 ◦ C (%) are given in Table 3. All compounds decomposed in one step. The thermal stabilities of the new cyclotriphosphazene derivatives ( **3a** – **3d** ) are obviously higher than those of the Schiff bases ( **2a** – **2d** ) and cyclotriphosphazene (1). For example, while the decomposition temperature (T _dm_ ) of **2a** was 275 ◦ C, the T _dm_ value was 458 ◦ C for compound **3a** , as shown in Figure 8.

**Figure 9:**
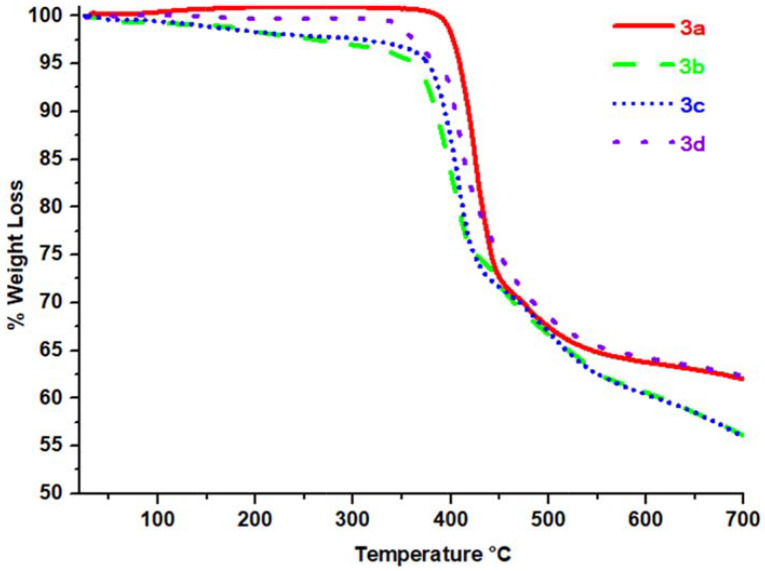
TGA curves of compounds **3a** – **3d** from 25 ◦ C to 700 ◦ C at a heating rate of 10 ◦ C/min under N2 flow of 50 mL/min.

The T _on_ and T _dm_ values of products decreased in the order of hydrogen ( **3a** ), bromine ( **3d** ), chlorine ( **3c** ), and fluorine ( **3b** ) derivatives of the Schiff bases, respectively (Table 3). The thermal stability of the products slightly reduced in the order of **3a** , **3d** , **3c** , and **3b** , as shown in Figure 9. The main factor affecting this trend is the size of the halogen group (Ph-X; X = F, Cl, Br) substituted on the Schiff base [45,46]. It is seen that as the size of the halogen atoms increases, the cross-linking process on decomposition decreases. Another factor explaining this trend is the increase of bond dissociation energy in the order of C-Br < C-Cl < C-F.

The char yields of new cyclotriphosphazene derivatives **3a** – **3d** (62.5% for **3a** ; 56.5% for **3b** ; 56.6% for **3c** ; 62.9% for **3d** ) are quite high because of the P-N skeleT _on_ and cross-linking process on decomposition (Table 3).

### 3.4. UV-Vis absorption properties of **3a** – **3d**

**Figure 10:**
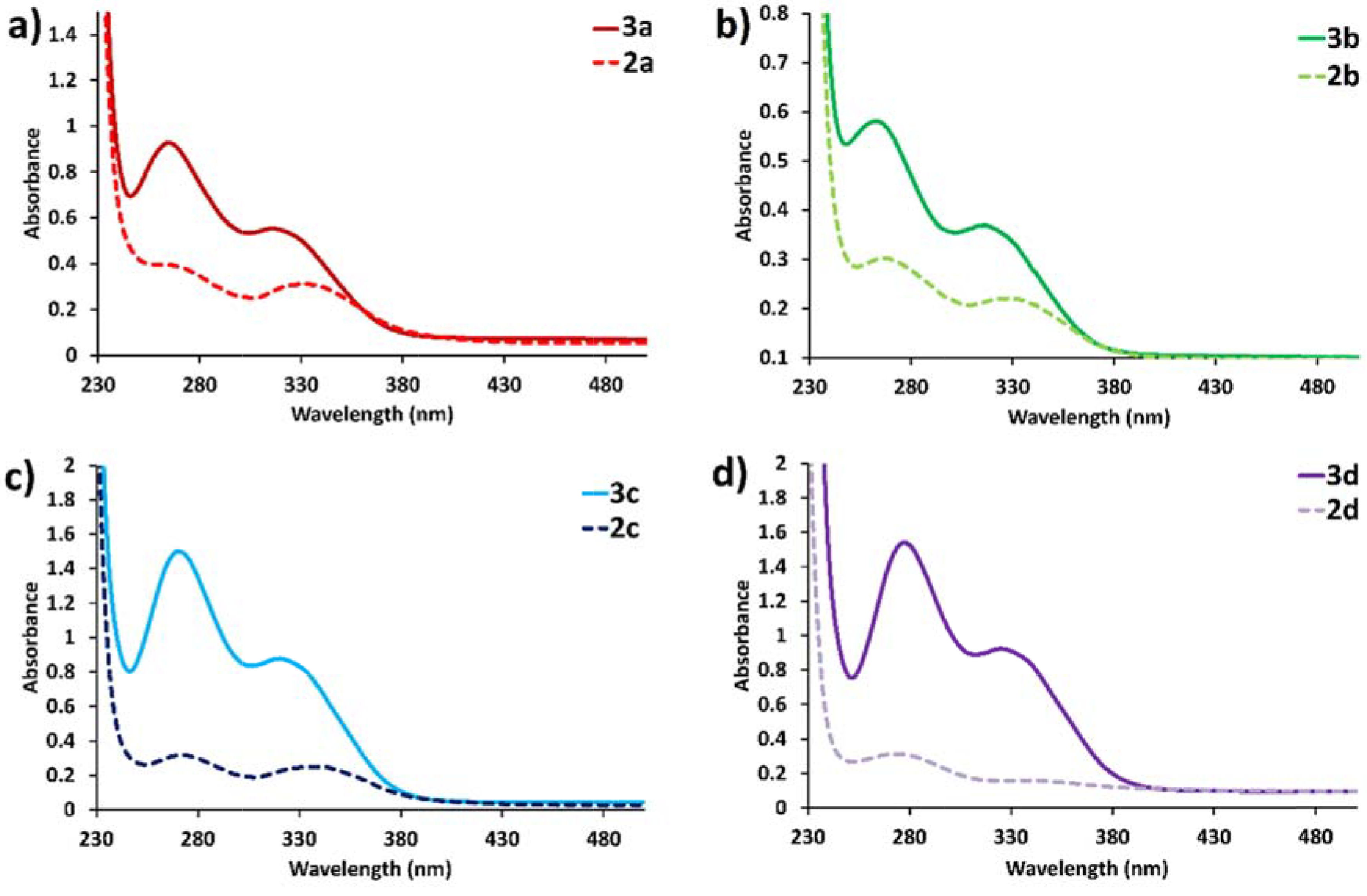
UV-Vis absorption spectra of 1 × 10 ^-5^ M **3a** – **3d** with their precursors **2a** – **2d** : a) **2a** , **3a** ; b) **2b** , **3b** ; c) **2c** , **3c** ; d) **2d** , **3d** in dichloromethane.

The optical properties of new cyclotriphosphazene derivatives ( **3a** – **3d** ) containing Schiff bases and precursor Schiff bases ( **2a** – **2d** ) were evaluated with UV-Vis spectroscopy. All of the spectral measurements were carried out in a spectroscopic quartz cuvette by using a micropipette at 25 ◦ C. UV-Vis electronic absorption spectra of compounds **2a** – **2d** and **3a** – **3d** were plotted in CH2 Cl2 and the epsilon values of all compounds in CH _2_ Cl _2_ were calculated and are given Table 4. As shown in Figure 10, 1 × 10 ^-5^ M of compounds **2a** – **2d** and **3a** – **3d** demonstrated the absorption maximum between 266–339 nm, attributed to π-π* transitions for benzene rings and azomethine moieties [47–50]. Importantly, the UV-Vis electronic absorption wavelengths of compounds **3a** – **3d** were similar to those of their precursor Schiff bases ( **2a** – **2d** ), which showed that there was no effective ground state interaction with the appended Schiff base on the cyclotriphosphazene scaffold. On the other hand, the absorbances of compounds **3a** – **3d** were much higher than those of the corresponding Schiff bases ( **2a** – **2d** ) at the same concentration, which was most probably due to the increasing number of absorbing Schiff base groups on the cyclotriphosphazene scaffold [51]. The molar absorption coefficients of **3a** – **3d** were as high as 10 ^4^ to 10 ^5^ L mol ^-1^ cm ^-1^ , which were determinative for π-π* state transition characteristics of cyclotriphosphazene derivatives ( **3a** – **3d** ) [52]. These obtained results are quite reasonable because it is well known that cyclophosphazenes are optically inert in the UV-Vis region and their photophysical properties can be adjusted according to the appended moiety [10,53–55].

**Table 4:** Absorption properties of compounds **2a** – **2d** and **3a** – **3d** .

Compounds	λabs* (nm)	ε* (L mol ^-1^ cm ^-1^ )
**2a**	266, 333	38800
**2b**	266, 328	30200
**2c**	272, 338	31700
**2d**	274, 339	31300
**3a**	265, 318	92700
**3b**	266, 321	57500
**3c**	271, 324	139900
**3d**	278, 330	154000

*Values are given for dichloromethane.

**Figure 11:**
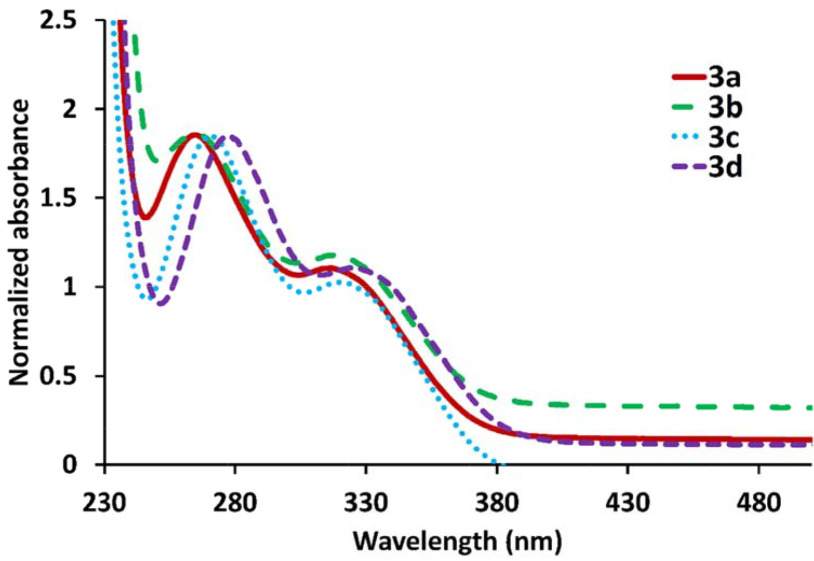
Normalized UV-Vis absorption spectra of **3a** – **3d** in dichloromethane.

The normalized UV-Vis electronic absorption spectra in CH _2_ Cl _2_ of compounds **3a** – **3d** are given in Figure 11. As can be seen, the maximum wavelengths of electronic absorption of compounds **3a** – **3d** changed for not only the first absorption bands (266–278 nm) but also the second absorption bands (318–330 nm) in the following order: **3d** > **3c** > **3b** > **3a** (Br > Cl > F > H). When the substitution at the Schiff base on the cyclotriphosphazene changed from H to Br, red shifts of the first and second electronic absorption bands were determined to be 13 nm and 22 nm, respectively. These alterations of the electronic absorption maxima of **3a** – **3d** can be attributed to the increase of polarizability of heavy atoms, which was consistent with previous reports in the literature [10,24,25].

The solvent system and working concentration can significantly affect the UV-Vis properties of molecules [24,56]. Therefore, solvent effect on absorption of compounds **3a** – **3d** were investigated with different solvents such as cyclohexane, 1,4-dioxane, THF, dichloromethane, DMSO, and water, as shown in Figure S13. Although water and cyclohexane were used for dissolution, the UV-Vis spectra of compounds **3a** – **3d** were not obtained due to the solubility problem of the compounds. As can be seen from Figure S13, compounds **3a** – **3d** have good solubility in organic solvents (1,4-dioxane, THF, dichloromethane) and moderate solubility in a polar solvent (DMSO). In addition, it can be understood that the UV-Vis electronic absorption of compounds **3a** – **3d** was not affected by the change of solvents and π-π* transitions between 266 and 330 nm were consistent without any shift. The effects of concentration on the UV-Vis electronic absorption spectra of compounds **3a** – **3d** were examined at various concentrations between 10 ^-5^ and 10 ^-6^ M, as shown in Figures S14–S17. The UVVis absorbance of compounds **3a** – **3d** gradually decreased without any red or blue shift when concentrations of solutions were diluted from 1 × 10 ^-5^ to 1 × 10 ^-6^ M, which indicated that no inter- or intramolecular interaction existed between attached Schiff base groups on the cyclotriphosphazene scaffold.

After evaluation of the UV-Vis electronic absorption properties of cyclotriphosphazene derivatives ( **3a** – **3d** ) containing Schiff bases, the fluorescence properties of compounds **3a** – **3d** were investigated at various concentrations between 10 ^-5^ and 10 ^-6^ M in 1,4-dioxane, THF, dichloromethane, and DMSO, which were excited between 250 and 350 nm. However, compounds **3a** – **3d** showed no appreciable fluorescence properties in these conditions. These results obtained for the fluorescence properties of Schiff base derivatives are expected and consistent with the literature because it is well known that C=N isomerization is the predominant decay process of the excited states for Schiff base derivatives with an unbridged C=N structure such that those compounds are often nonfluorescent [56–60].

## 4. Conclusions

To summarize, we report on the synthesis and characterization of a series of cyclotriphosphazene derivatives containing Schiff base ligands and their thermal and absorbance properties. It was found that the newly synthesized compounds ( **3a** – **3d** ) have good thermal properties, making them potentially suitable for some industrial applications, such as flame-retardant additives to polymers. It was also found that the electronic absorption maxima of **3a** – **3d** are slightly shifted to the red region when the substitution at the Schiff base on cyclotriphosphazene changes from H to Br.
